# COVID-19’s Impact on Digital Health Adoption: The Growing Gap Between a Technological and a Cultural Transformation

**DOI:** 10.2196/38926

**Published:** 2022-09-19

**Authors:** Bertalan Meskó

**Affiliations:** 1 The Medical Futurist Institute Budapest Hungary

**Keywords:** COVID-19, digital health, future, cultural transformation, medical information, technology adoption, health care, physician burnout, burnout

## Abstract

Health care in the 21st century has started undergoing major changes due to the rising number of patients with chronic conditions; increased access to new technologies, medical information, and peer support via the internet; and the ivory tower of medicine breaking down. This marks the beginning of a cultural transformation called digital health. This has also led to a shift in the roles of patients and medical professionals, resulting in a new, equal partnership. When COVID-19 hit, the adoption of digital health technologies skyrocketed. The technological revolution we had been aiming for in health care took place in just months due to the pandemic, but the cultural transition is lagging. This creates a dangerous gap between what is possible technologically through remote care, at-home lab tests, or health sensors and what patients and physicians are actually longing for. If we do it well enough now, we can spare a decade of technological transformations and bring that long-term vision of patients becoming the point of care to the practical reality of today. This is a historic opportunity we might not want to waste.

## Introduction

Health care in the 21st century has been going through major changes due to the rising number of patients with chronic conditions; increased access to new technologies, medical information, and peer support via the internet; and the disintegration of the ivory tower of medicine [1]. A cultural transformation called digital health has begun [1].

This has also led to a shift in the roles of patients and medical professionals. The role of the passive patient who only turns to physicians after a symptom arises, has been changing into a proactive, empowered role with a desire to be involved in their care. These “empowered patients” (e-patients) are equipped with technologies and information, they are experts in their health or disease management, and they use electronic devices to measure data [2]. Similarly, the role of a burnt-out physician spending half of their time with administrative tasks has been changing into an “empowered physician” (e-physician) role where they are guides for their patients in the jungle of digital information instead of being the keyholders to the ivory tower of medicine [3].

The new roles have started breaking down the status quo too, that is, the hierarchical relationship between patients and medical professionals. In its place is a new, equal partnership. To put it simply, patients have become the newest members of the medical team [4].

However, the biggest shift in this transformation is about digital health technologies making patients the point of care, receiving diagnosis or treatment wherever they are. Health sensors and portable diagnostic devices measuring fitness activities, sleep quality, electrocardiography, or blood pressure have allowed patients to become further involved in their care by providing them with data that were previously only accessible within the ivory tower [5].

It seems that technologies are becoming available at an unprecedented rate and that the cultural transformation is the component that will take time. Learning to deal with equal partnership takes more time than learning to use a sensor or smartwatch. Regardless, if the technological and cultural transformations can take place almost simultaneously, there is a big chance that the core elements of care such as empathy, compassion, and relationships based on trust will remain intact.

## COVID-19’s Impact on Digital Health

When COVID-19 hit, the adoption of digital health technologies skyrocketed. It was a necessity, not a choice. Still, telemedical applications and services, health sensors, 3D printed protective equipment, and at-home laboratory tests have become part of everyday care in just weeks in March and April 2020.

In Catalonia, Spain, telemedicine took the place of face-to-face primary care visits in less than a month. While around 18,000 telemedical and 150,000 face-to-face visits were conducted in early March 2020, the number of telemedical visits rose to over 100,000 and the number of face-to-face visits decreased to 21,000 just 4 weeks later [6]. In the United States, appointments on telemedicine services such as PlushCare and Amwell increased by 70% and 158%, respectively.

After remote care, remote testing was the next major disruptor. Waiting in lines to take a biological sample meant a risk of exposure to infection. Wherever it was possible, at-home lab tests were prioritized. COVID-19 antigen and antibody tests appeared on the market in addition to companies offering direct-to-consumer blood test sampling and analysis. The sample collection for many tests from food allergy to genetic analyses started taking place in patients’ homes.

The pandemic has had a lasting toll on mental health. The meditation smartphone app Headspace has seen a greater than 500% increase in inbound interest from companies seeking mental health help for their employees [7]. The number of users starting its “stressed meditation” offering increased by 6 folds.

Disinfectant robots started roaming hospital floors, reducing people’s risk of infection. Do-it-yourself groups around the world started producing 3D printed materials such as medical tools, protective equipment, and practically anything else needed when traditional production or supply was scarce and health institutions were overwhelmed.

Artificial intelligence (AI) has taken the central stage too [8]. The first report about a potential outbreak in Wuhan, China, came from a Canadian start-up called BlueDot. It used a machine learning algorithm to sift through news reports, airline ticketing data, and reports of animal disease outbreaks to detect public health trends and dangers. AI has also been used to organize supply chains; sort out ventilators in a country; find new drug combinations that could treat sick patients through network science; analyze, monitor, screen, and triage patients with COVID-19 to support hospitals with resource allocation; or facilitate drug discovery and vaccine development. Researchers at the Massachusetts Institute of Technology even developed an AI-based voice analyzer to identify asymptomatic patients with COVID-19 from cough recordings on their smartphones.

Even before the pandemic, digital health investments were steadily increasing year by year; however, 2020 was a record-breaking year. Venture funding for the sector shot up 66% over 2019, with a record $14.8 billion raised globally, according to Mercom Capital Group [9]. Telemedicine, of course, was the leading investment target, receiving $4.3 billion in venture capital funding in 2020 [9].

Needless to say, digital health has seen an unprecedented rate of adoption. However, clinical reality does not reflect this optimism. Health care is overwhelmed worldwide, physicians rapidly burn out under immense pressure, patients with chronic conditions lack access to care, treatments get delayed, and medical professionals do their best to maintain the system. There are not many resources left to innovate.

## The Lagging Cultural Transformation

Many examples have shown that the use of technologies does not automatically lead to better care [10]. Family members have to use a telemedical robot equipped with a tablet device to communicate with their loved ones in the hospital. Even some end-of-life discussions had to take place through telemedical robots [11]. Without proper guidance, such use of an advanced technology can lead to mental health issues for families later on.

Recent papers (eg, Ritchey et al [12]) concluded that although technology does not replace face-to-face encounters, it can offer meaningful connection; such an experience requires redefining the traditional palliative care model. Caregivers and family members have to learn to live with the constant fear that technology might fail and have to give themselves permission to make mistakes while they learn a new care model [12].

Another challenge that was amplified due to the rise of technologies and access to information was the fight against misinformation about COVID-19 and vaccination. Antivaccination groups make it harder to vaccinate enough people to leave the pandemic behind [13]. It has also indicated how important a trustful and strong medical and scientific leadership is.

## Discussion

The technological revolution we had been aiming for in health care took place in just months due to the pandemic, but the cultural transition is lagging. This creates a dangerous gap between what is possible technologically through remote care, at-home lab tests, or health sensors and what patients and physicians are really longing for. Based on our previous studies [2,3], it is empathy, attention, and time, not AI or more health sensors.

The idealistic vision of digital health is to allow patients to have meaningful conversations with medical professionals while being surrounded and supported by advanced, seamless, and almost invisible technologies.

It usually takes a few months to adopt a new habit. We will have been living with masks, social distancing, and remote care for so long by the time the pandemic ends that we might never go back to the old “norm” [14]. Additionally, once most patients realize they have a choice between getting the required information virtually in minutes or in person by traveling and waiting for hours, all the while increasing their risk of exposure to infections, they might never want to go back.

This new kind of e-patient and e-physician, who are reimbursed for virtual visits, could stay with us indefinitely [15]. Therefore, we, patients and health care professionals alike, have to find a way to live up to this new norm emotionally, mentally, and culturally.

Certain efforts have been demonstrated to help ease this process: health information campaigns launched by governments [16], medical associations providing guidance on using digital health technologies [17], medical curriculums designed to prepare students for working with e-patients [18], and policies that support remote care services and consider them the new norm [19].

If we do it well enough now, we can spare a decade of technological transformations and bring that long-term vision of patients becoming the point of care to the practical reality of today. This is a historic opportunity we might not want to waste.

## Abbreviations

AI: artificial intelligence
e-patient: empowered patient
e-physician: empowered physician
